# Papillon–Lefèvre syndrome: a series of five cases among siblings

**DOI:** 10.1186/s13256-016-1051-z

**Published:** 2016-09-22

**Authors:** Zyad M. AIBarrak, Adel S. Alqarni, Elna P. Chalisserry, Sukumaran Anil

**Affiliations:** 1 King Abdulaziz Medical City, Ministry of Health, Riyadh, Saudi Arabia; 2 Department of Preventive Dental Sciences, College of Dentistry, Prince Sattam Bin Abdulaziz University, 153, AIkharj, 11942 Riyadh Saudi Arabia; 3 Department of Maxillofacial Surgery and Diagnostic Sciences, College of Dentistry, Jazan University, Jazan, 82943 Saudi Arabia

**Keywords:** Papillon–Lefèvre syndrome, Consanguinity, Periodontitis, Premature tooth loss, Cathepsin C, Gene mutation, Hyperkeratosis, Palmoplantar keratosis

## Abstract

**Background:**

Papillon–Lefèvre syndrome is a rare autosomal recessive disorder characterized by palmoplantar hyperkeratosis and aggressively progressing periodontitis leading to premature loss of deciduous and permanent dentition. The etiopathogenesis of the syndrome is relatively obscure, and immunologic, genetic, or possible bacterial etiologies have been proposed.

**Case presentation:**

A series of five cases of Papillon–Lefèvre syndrome among the siblings in a family is presented here: a 3-year-old Arab girl, a 4-year-old Arab boy, a 11-year-old Arab boy, a 12-year-old Arab boy, and a 14-year-old Arab boy. The patients presented with severe gingival inflammation and mobility of teeth. The clinical manifestations were typical of Papillon–Lefèvre syndrome and the degree of involvement of the oral and skin conditions varied among them.

**Conclusions:**

This case series stresses the consanguinity in the family as an etiologic factor. All siblings in the family were affected with Papillon–Lefèvre syndrome which makes this a rare case. A multidisciplinary approach with the active participation of a dental surgeon, dermatologist, and pediatrician is essential for the management of cases of Papillon–Lefèvre syndrome.

## Background

Papillon–Lefèvre syndrome (PLS) is a rare autosomal recessive heterogeneous disorder, which is characterized by palmoplantar hyperkeratosis, early loss of primary and permanent teeth, and associated calcification of the dura mater [1]. The onset of disease usually coincides with the eruption of primary teeth. Boys and girls are equally affected, with no racial predominance [2]. The onset of the cutaneous lesion of PLS may appear at birth or at 1 to 2 months of age, but most commonly appears between the age of 6 months and 4 years which coincides with the eruption of primary teeth [3]. Associated features may include intracranial calcifications, susceptibility to bacterial infections, and mental retardation [4].

The exact etiology of PLS is still obscure; however, microbiologic, immunologic, and genetic factors have all been linked to the development of the syndrome. The disorder can be hereditary, acquired, or associated with other syndromes. PLS is autosomal recessive, and consanguinity has been demonstrated in 20 to 40 % of patients [5]. Earlier reports have described PLS in children of consanguineously married parents [6, 7]. If both parents are carriers of the defective gene there is a 25 % risk for their children to be affected [8].

Consanguineous marriage is a cultural practice with ancient roots, and 20 % of the world’s population currently lives in communities that prefer this form of marriage [9]. Arab countries have the highest rates (20 to 50 %) of consanguineous marriage in the world [10]. An etiological link to *Cathepsin C* (*CTSC*) gene mutations leading to a deficiency of cathepsin C enzymatic activity has been identified [11, 12]. *Aggregatibacter actinomycetemcomitans* was reported to have a significant role in the progression of periodontal involvements. Other microbial agents including *Porphyromonas gingivalis*, *Fusobacterium nucleatum*, and *Treponema denticola* have also been suggested to have causal effects [13]. PLS is characterized by aggressively progressive periodontitis accompanied by palmoplantar hyperkeratosis. In some cases, the hyperkeratosis may spread to the knees, elbows, back, and fingers [1]. Disorders such as abscesses of the skin, liver, kidneys, and brain, as well as dural calcification have also been reported [14, 15].

The oral findings of PLS are hypermobility, drifting, migration, and exfoliation of teeth without any signs of root resorption. The periodontitis causes premature loss of deciduous and permanent teeth, often leaving the patient edentulous in adolescence [16]. The gingiva gets inflamed with the eruption of the primary teeth. Subsequently a rapid destruction of periodontium occurs and most affected children experience premature loss of their primary teeth. The gingiva resumes normal appearance after exfoliation of the primary dentition. The aggressive inflammatory periodontal process then re-triggers itself after the eruption of the permanent teeth, and in general all or most of the permanent dentition is lost during the teenage years [7]. Radiographic features are characterized by generalized loss of alveolar bone [3] and intracranial calcification [17]. A well-documented case series of PLS among five siblings in a family is presented. The oral and cutaneous manifestations and current treatment modalities are discussed.

## Case presentation

**Fig. 1:**
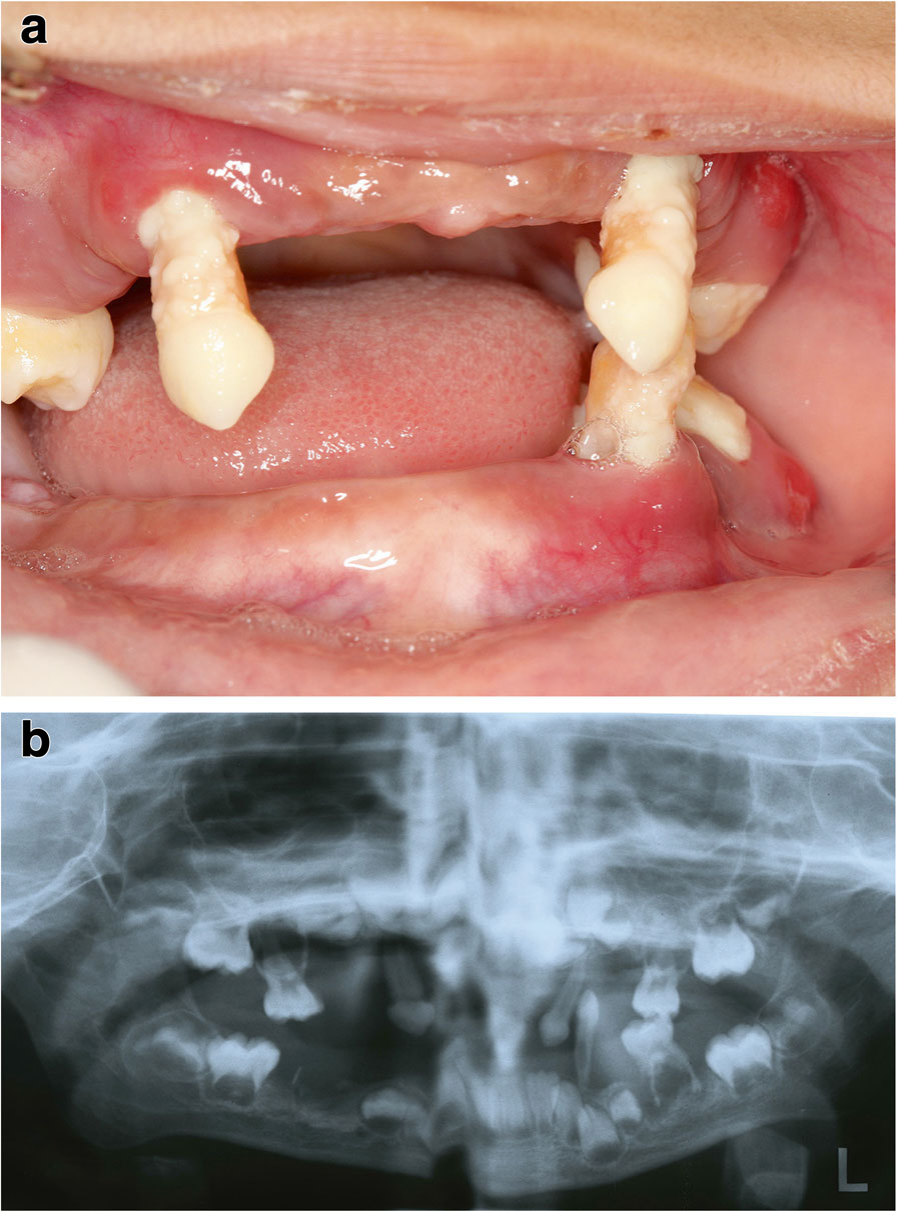
Case 1. **a** Clinical appearance of the deciduous teeth, note the exfoliated primary teeth, gingivitis, and plaque accumulation. **b** Panoramic radiograph showing bone loss and migrated teeth with bone loss

**Fig. 2:**
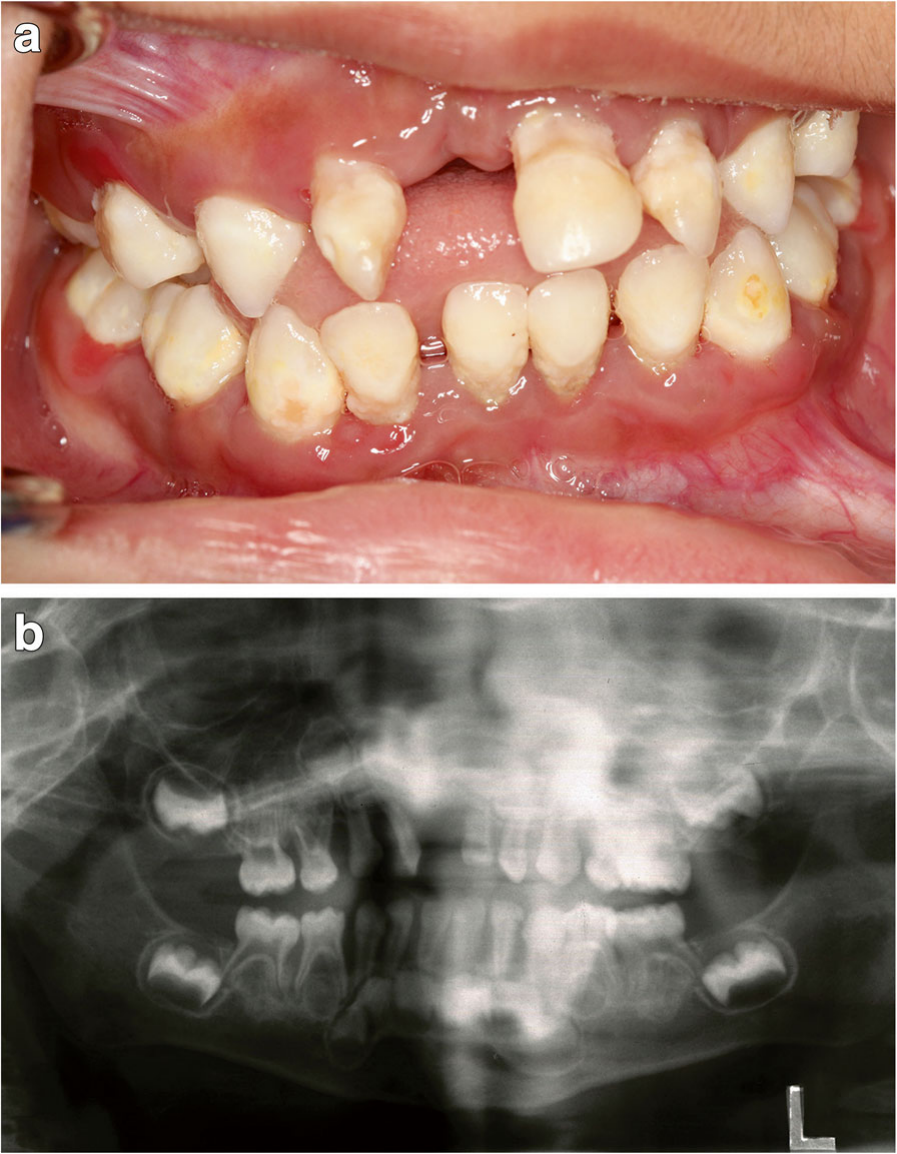
Case 2. **a** Intraoral appearance with gingival inflammation, plaque accumulation, migration of teeth. **b** Panoramic radiograph showing bone destruction and interdental spacing

**Fig. 3:**
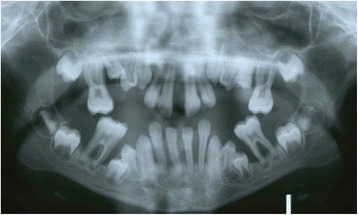
Case 3. Panoramic radiograph showing severe periodontal destruction and migration of teeth

**Fig. 4:**
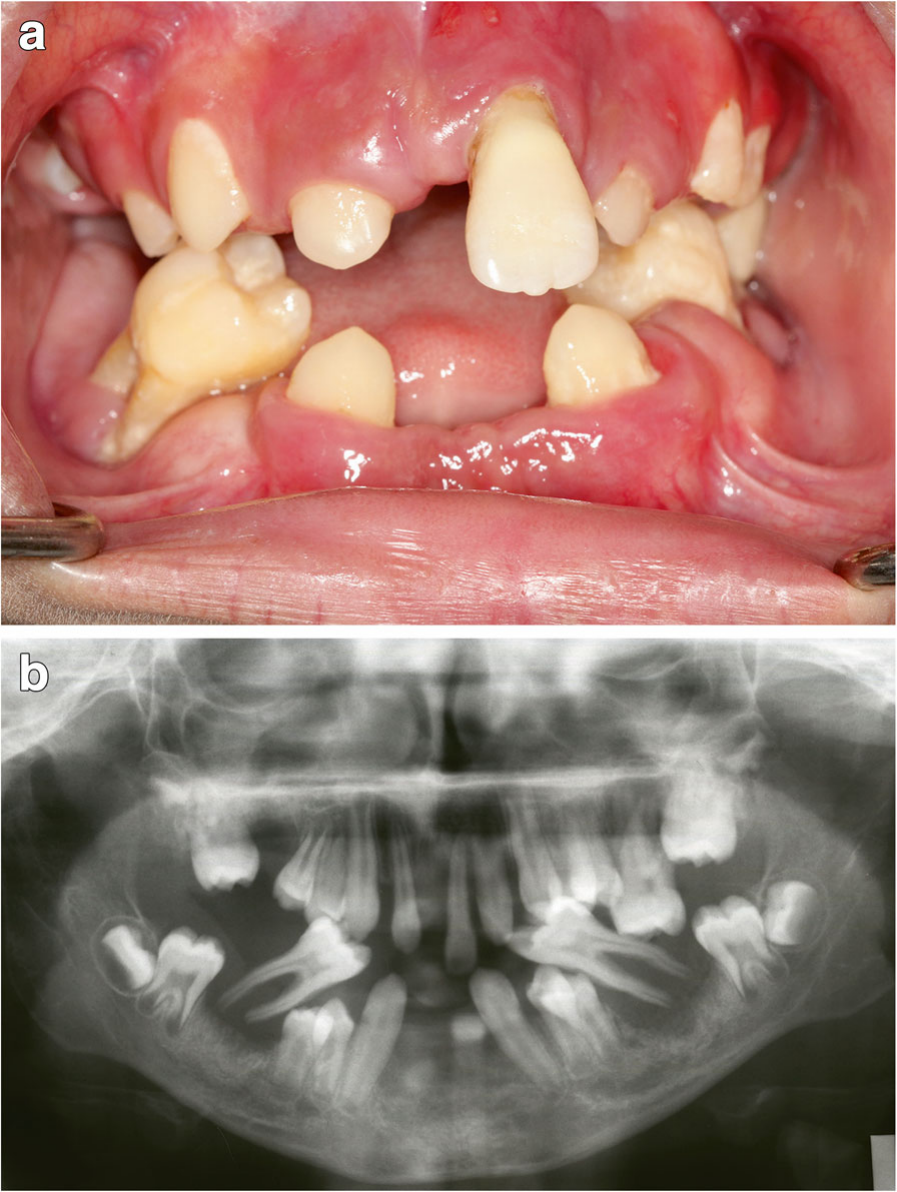
Case 4. **a** Severe gingivitis with periodontal destruction and migration of permanent molars. **b** Panoramic radiograph showing severe periodontal destruction, note the migration and floating of the lower first molars

**Fig. 5:**
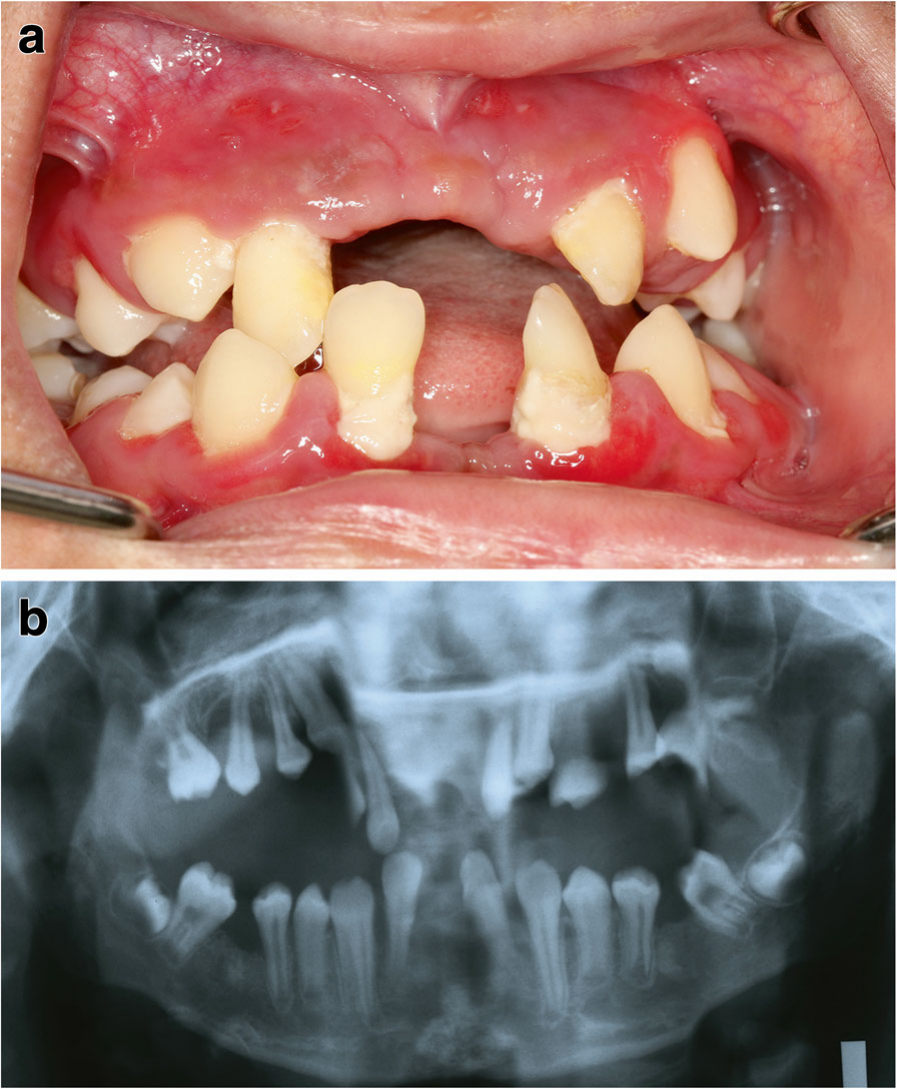
Case 5. **a** Intraoral appearance with loss of permanent anterior from both jaws, severe inflammation, and enlargement of the gingiva. **b** Panoramic radiograph showing severe destruction of the alveolar bone and loss of permanent anterior teeth

**Fig. 6:**
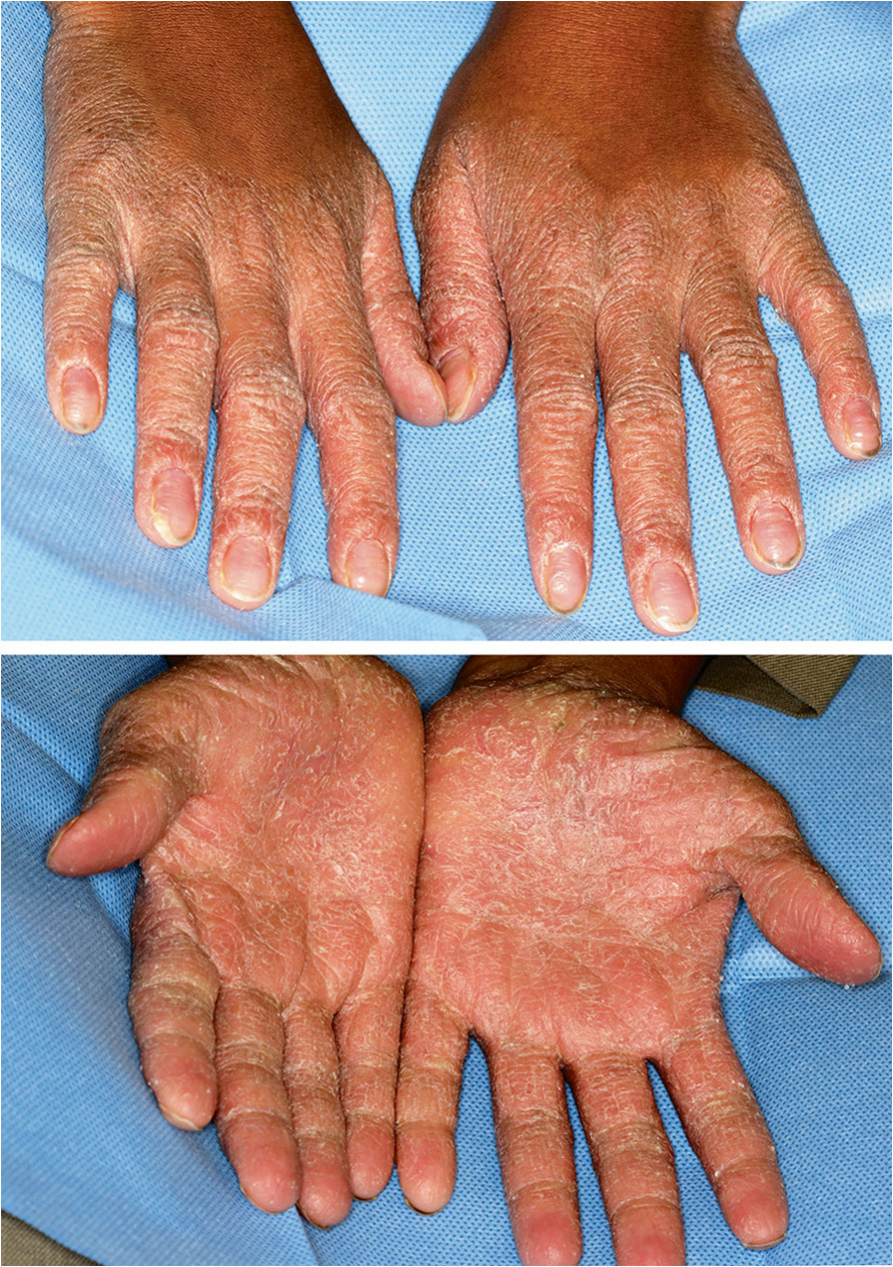
Hyperkeratotic lesions on the palms and the dorsal surface (Case 5)

**Fig. 7:**
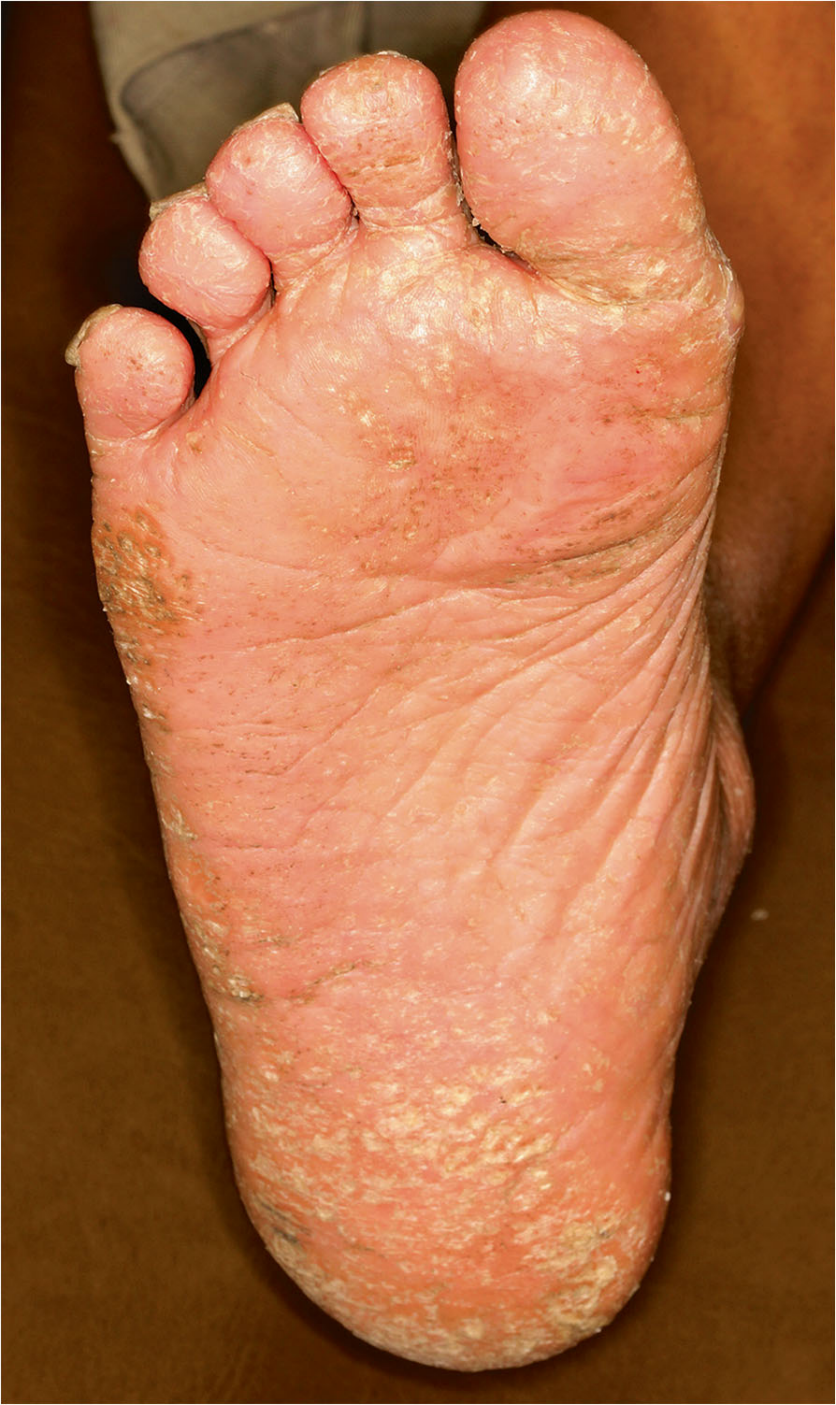
Hyperkeratotic lesions on the soles (Case 5)

The case series were seen at an out-patient department. The clinical manifestations were typical of PLS and the degree of involvement of the oral and skin conditions varied among the siblings (Figs. 1, 2, 3, 4, 5, 6, and 7). The patient details and clinical features are depicted in Table 1. The parents of these five children are first cousins and the possibility of consanguinity was established as a probable etiologic factor. Since all the patients were otherwise healthy and their medical records did not show any hematological abnormalities, the cases were referred to the King Faisal Specialty Hospital, Riyadh, Saudi Arabia. The center has a unit for managing cases of PLS, which coordinates dental, dermatology, and genetic disorder research and management due to the high number of cases in Saudi Arabia. It acts as a national coordination unit and gives advice and follow up on cases of PLS.

**Table 1:** Patient details and clinical findings

Variables		Cases				
		1	2	3	4	5
Gender		F	M	M	M	M
Age (years)		3	4	11	12	14
Periodontal manifestations	Gingivitis	+	+	+	+	+
	Periodontitis; Basic Periodontal Examination (BPE) Score	4	4	4	4	4
	Primary teeth loss	+	+	+	+	+
	Permanent teeth loss	–	–	–	+	+
	Alveolar bone resorption	+	+	+	+	+
	Halitosis	+	+	+	+	+
Skin manifestations	Palmoplantar hyperkeratosis	–	+	+	+	+
	Elbows	+	+	+	+	+
	Knees	+	+	+	–	+
	Toes	+	+	+	–	–
	Dorsal fingers	–	*–*	+	+	–

+ present, − absent, *F* female, *M* male

Early extraction of periodontally involved permanent teeth has been considered to be a mode of treatment to preserve alveolar bone [18, 19]. Treatment modalities other than extraction have been attempted in patients with PLS. Local debridement and/or systemic antibiotics alone or in combination have shown transient improvement of the condition [20]. Since there is no definitive treatment for cases of PLS, rehabilitation of the dentition is done considering factors such as age and psychosocial impact.

### Case 1

A 3-year-old Arab girl was examined as part of an investigation of her eldest brother’s case. All her anterior and most of her posterior primary teeth were lost. There was inflammation of her gingiva with plaque accumulation in her teeth (Fig. 1 and Table 1). Her palms and soles appeared normal. However, thickening of the skin was observed in her knees, elbows, and toes. Plaque was present in almost all her remaining deciduous teeth. Basic Periodontal Examination (BPE) using World Health Organization (WHO) 621 probe showed a code 4 in her remaining teeth [21]. Scaling was performed in our clinic and her parents were advised to maintain her oral hygiene. Home care measures were emphasized. Temporary space maintainers were fabricated and periodic follow up was advised.

### Case 2

A 4-year-old Arab boy presented to our clinic with exfoliated maxillary right central and lateral incisor. Gingivitis and plaque accumulation were present in his remaining teeth. The BPE showed a code between 3 and 4 in his remaining teeth. There was alveolar bone destruction around all erupted and erupting dentition. Keratosis of his palms and soles was present at a mild degree (Fig. 2 and Table 1). Scaling was performed in our clinic. We advised that he had periodic oral hygiene measures. A temporary denture was fabricated to wear during the daytime.

### Case 3

An 11-year-old Arab boy presented with mobile protruded and migrated maxillary and mandibular anterior teeth. All his primary teeth were lost. There was severe bone destruction around his permanent teeth. His molars were all mobile with less than one third bone support. A BPE code 4 was recorded in all his molars and incisors. There was bleeding from his gingiva with halitosis (Fig. 3 and Table 1). He had dermatologic manifestations such as keratinized skin in his joints, palms, and soles. Scaling was done in our clinic to remove all debris, plaque, and calculus. Periodic scaling (monthly) was advised and strict oral care measures were advised.

### Case 4

A 12-year-old Arab boy presented with multiple exfoliated teeth. His oral hygiene was relatively better with gingival enlargement around erupting teeth. Most of his permanent anterior teeth were lost with severe bone destruction around his remaining teeth (Fig. 4, Table 1). A periodontal examination recorded a score of 4 (deep pocket) in his molars and incisors. His lower molars appeared floating without any bone support. There was severe palmar plantar keratosis with keratinization of the dorsal surface of his hands. His molars were extracted, transitional dentures were given, and a follow-up regimen was advised.

### Case 5

A 14-year-old Arab boy, the eldest brother of these patients, was the one who presented for treatment: the replacing of his anterior teeth which were exfoliated. On radiographic examination severe bone destruction was noticed around his remaining teeth (Fig. 5). Periodontal recording using the BPE index showed a score of 4 for most of his remaining teeth. Associated dermatologic findings were conclusive of PLS, such as sever palmar plantar keratosis which affected the dorsal surface of his palms (Figs. 6 and 7, Table 1). Scaling and root planning was performed in his first visit and he was kept on a strict oral hygiene regimen. A temporary partial denture was fabricated and he was scheduled for implant therapy at a later stage.

## Discussion

The etiopathogenesis of the syndrome is relatively obscure and immunologic, genetic, or possible bacterial etiologies have been proposed [22]. The incidence in Saudi Arabia is higher compared with other parts of the world which may be attributed to cluster marriages [2, 3]. The severe periodontal destruction seen in PLS may be the result of loss of function mutation in the *CTSC* gene resulting in the dysregulation of localized polymorphonuclear leucocytes in the periodontal tissues [23].

One third of the cases of PLS reported in the literature had consanguineous parents [8, 16, 24]. PLS shows an autosomal recessive pattern and there is 25 % chance for the offspring getting affected from phenotypically healthy parents who carry the autosomal gene [25]. In the present case series all the children (five siblings) were affected which makes this a rare case. Most of the cases reported so far had two siblings affected [7, 26–28] except for a few cases in which three or four siblings were affected [29–33]. Mutations in the *CTSC* gene have been reported to result in PLS and the complete absence of cathepsin C activity is required in order to develop the clinical phenotype of PLS [25].

A possible bacterial etiology has also been proposed and it is believed that *Aggregatibacter actinomycetemcomitans, Porphyromonas gingivalis, Fusobacterium nucleatum*, and *Prevotella intermedia* may be among the organisms involved in periodontal breakdown [34]. General periodontal treatment modalities usually fail in patients with PLS, and the rapid progression of periodontitis results in severe loss of alveolar bone [35, 36]. Treatment modalities such as systemic and local antibiotic treatment, and synthetic retinoids have been tried with limited success [37]. Prolonged use of oral retinoids has been shown to be beneficial in preventing exfoliation of permanent teeth in children [38]. The rapid destruction of alveolar bone around the primary and permanent teeth results in atrophic jaws. Hence, the oral rehabilitation of these cases is challenging and implant-supported overdentures are generally recommended in edentulous patients with PLS [39].

## Conclusions

- PLS is an autosomal recessive genetic disorder characterized by palmoplantar hyperkeratosis associated with severe early-onset periodontitis and premature loss of primary and permanent teeth.
- Even though the occurrence of PLS among siblings is documented this is the first case where all five siblings were affected.
- There is no definitive treatment for PLS cases. Symptomatic management is followed.
- A multidisciplinary approach with the active participation of a dental surgeon, dermatologist, and pediatrician is essential for the management of case of PLS.
